# Supplementary material for: Spatial and Temporal Distribution Characteristics and Potential Risks of Sulfonamides in the Shaanxi Section of the Weihe River
Source: Int J Environ Res Public Health. 2022 Jul 15;19(14):8607. doi: 10.3390/ijerph19148607 (PMC9323655; doi:10.3390/ijerph19148607)
Supplement: Supplementary file 1 [file ijerph-19-08607-s001.zip › ijerph-1777641-supplementary.pdf]

Table S1. Mainstream and main tributaries of the Shaanxi section of the Weihe River.

Number	River Name	Length/km	Area/km^2
1	Weihe River	502.4	67108
2	Qianhe River	162.6	3493
3	Shitouhe River	77.5	676.02
4	Jinghe River	272	9246
5	Qishuihe River	151	3824
6	Heihe River	125.8	2258
7	Shichuanhe River	137	4478
8	Laohe River	43.8	346
9	Fenghe River	78	1386
10	Bahe River	109	2581
11	Luohe River	680.3	26905
